# Effects of Hypoxia and Bed Rest on Markers of Cardiometabolic Risk: Compensatory Changes in Circulating TRAIL and Glutathione Redox Capacity

**DOI:** 10.3389/fphys.2018.01000

**Published:** 2018-07-30

**Authors:** Gianni Biolo, Filippo G. Di Girolamo, Adam McDonnell, Nicola Fiotti, Filippo Mearelli, Roberta Situlin, Arianna Gonelli, Barbara Dapas, Mauro Giordano, Mitja Lainscak, Gabriele Grassi, Giorgio Zauli, Paola Secchiero, Igor Mekjavic

**Affiliations:** ^1^Clinica Medica, Department of Medical, Surgical and Health Sciences, University of Trieste, Trieste, Italy; ^2^Department of Automation, Biocybernetics and Robotics, Jozef Stefan Institute, Ljubljana, Slovenia; ^3^Dipartimento di Morfologia, Chirurgia e Medicina Sperimentale, University of Ferrara, Ferrara, Italy; ^4^Department of Life Science, University of Trieste, Trieste, Italy; ^5^Ospedale Clinicizzato di Marcianise, Dipartimento di Scienze Mediche, Chirurgiche, Neurologiche, Metaboliche e dell’Invecchiamento, Università degli Studi della Campania Luigi Vanvitelli, Marcianise, Italy; ^6^Department of Internal Medicine, General Hospital Murska Sobota and Faculty of Medicine, University of Ljubljana, Ljubljana, Slovenia

**Keywords:** hypoxia, TRAIL, glutathione, oxidative stress, bed rest, omega-3 fatty acids

## Abstract

In chronic diseases, hypoxia and physical inactivity are associated with atherosclerosis progression. In contrast, a lower mortality from coronary artery disease and stroke is observed in healthy humans residing at high altitude in hypoxic environments. Eleven young, male volunteers completed the following 10-day campaigns in a randomized order: hypoxic ambulatory, hypoxic bed rest and normoxic bed rest. Before intervention, subjects were evaluated in normoxic ambulatory condition. Normobaric hypoxia was achieved in a hypoxic facility simulating 4000 m of altitude. Following hypoxia, either in bed rest or ambulatory condition, markers of cardiometabolic risk shifted toward a more atherogenic pattern consisting of: (a) lower levels of total HDL cholesterol and HDL2 sub-fraction and decreased hepatic lipase; (b) activation of systemic inflammation, as determined by C-reactive protein and serum amyloid A; (c) increased plasma homocysteine; (d) decreased delta-5 desaturase index in cell membrane fatty acids, a marker of insulin sensitivity. Bed rest and hypoxia additively decreased total HDL and delta-5 desaturase index. In parallel to the pro-atherogenic effects, hypoxia activated selected anti-atherogenic pathways, consisting of increased circulating TNF-related apoptosis-inducing ligand (TRAIL), a protective factor against atherosclerosis, membrane omega-3 index and erythrocyte glutathione availability. Hypoxia mediated changes in TRAIL concentrations and redox glutathione capacity (i.e., GSH/GSSG ratio) were greater in ambulatory conditions (+34 ± 6% and +87 ± 31%, respectively) than in bed rest (+17 ± 7% and +2 ± 27% respectively). Hypoxia-induced cardiometabolic risk is blunted by moderate level of physical activity as compared to bed rest. TRAIL and glutathione redox capacity may contribute to the positive interaction between physical activity and hypoxia.

**Highlights**:

– Hypoxia and bed rest activate metabolic and inflammatory markers of atherogenesis.

– Hypoxia and physical activity activate selected anti-atherogenic pathways.

– Hypoxia and physical activity positive interaction involves TRAIL and glutathione.

## Introduction

The association of hypoxia with decreased physical activity is frequently observed in patients affected by chronic cardiopulmonary pathologies, such as COPD, pulmonary fibrosis, OSAS or heart failure. Most of these conditions are characterized by excess mortality due to atherosclerosis (Roversi et al., 2014). In contrast to patients with chronic hypoxic diseases, a lower mortality from coronary heart disease and stroke is observed in humans residing at high altitude in hypoxic environments (Faeh et al., 2009). While the link between physical inactivity and cardiovascular risk is well established (Biolo et al., 2005a); hypoxia can activate both pro- and anti-atherogenic pathways, involving the immune response, redox balance, lipid metabolism and insulin sensitivity (Faeh et al., 2009; Roversi et al., 2014). Despite the fact that the combination of hypoxia and reduced physical activity is so frequently observed in patients, the interaction between these mechanisms in influencing cardiometabolic risk has been scarcely investigated. In fact, the specific effects of inactivity and hypoxia are difficult to separate from confounding variables arising from diseases. Healthy volunteers confined in a hypoxic facility, investigated during enforced bed rest or in ambulatory condition, provided an appropriate model to investigate the separated and combined effects of normobaric hypoxia and muscle unloading on development of cardiovascular risk (Debevec et al., 2014). We have selected a number of metabolic (circulating and cell membrane lipids, reverse cholesterol transport enzymes, homocysteine), immune (circulating acute phase proteins) and redox (intracellular glutathione status and kinetics) biomarkers linked to atherosclerosis (Ashfaq et al., 2006; Sasaki et al., 2012; Watkins and Hotamisligil, 2012; Gortan Cappellari et al., 2013; Rong et al., 2017) and potentially affected by decreased oxygen availability and/or muscle unloading (Blaise et al., 2005; Biolo et al., 2008; Bosutti et al., 2008; Mazzucco et al., 2010a; Bruning et al., 2012). In addition, we have determined the circulating levels of TRAIL (Biolo et al., 2012), which are also potentially upregulated by oxygen availability (Huang et al., 2011; Liu et al., 2015). Increased circulating TRAIL levels are associated with decreased systemic inflammation (Voltan et al., 2016), improved endothelial functions and downregulation of atherosclerosis (Forde et al., 2016).

## Materials and Methods

### Study Design

Our investigation was performed within the frame of the 10-day hypoxic bedrest study project ‘LunHab: Lunar Habitat Simulation’ (Debevec et al., 2014). The experimental protocol was conformed to the Declaration of Helsinki and following amendments and was approved by the National Committee for Medical Ethics at the Ministry of Health of the Republic of Slovenia (205/2/11). The overall study design was previously described (Debevec et al., 2014). Briefly, eleven young male volunteers (age 24 ± 4 years; body mass index 22 ± 2 kg/m^2^) were selected to participate in the experimental 10-day hypoxic bedrest study at the Olympic Sport Centre in Planica (Ratece, Slovenia). All enrolled volunteers were physically active before the study and none of them was under medication. After obtaining a written informed consent from each volunteer, all subjects underwent three experimental campaigns: (a) normoxic bed rest (inspired partial pressure of oxygen: 21 kPa); (b) hypoxic bed rest (inspired partial pressure of oxygen: about 12.5 kPa, simulating ambient elevation at about 4000 m above sea level); (c) hypoxic ambulatory. Every campaign included 5-day pre-intervention period, during which the volunteers were housed at the Olympic Sport Centre, allowed them to adapt to the facility and to the prescribed diet. Participants were allowed to move freely in the experimental area and were encouraged to engage in their usual daily physical activity routines as much as possible. Assessment of the exercise intensity was based on the questionnaire regarding daily physical activity that each participant filled out prior to the study. The exercise sessions during the ambulatory conditions were not meant to provide a training stimulus, but to mimic the level of daily activity. During the 10-day hypoxic ambulatory confinement, the participants also performed 2 low-intensity exercise sessions per day. Each exercise session comprised 30 min of stepping on a 30 cm step to induce an individually targeted heart rate (HR) corresponding to approximately 60% of maximal HR measured during a graded exercise test to exhaustion on a cycle ergometer. Each campaign lasted 20 days (5 days: dietary and environmental adaptation; 10 days: interventions; 5 days: recovery) and was separated from the others by 1-month wash-out period. Throughout the study periods, all subjects received six meals per day (3 main meals and 3 snacks). Before each campaign, individual resting energy expenditure was calculated according to the FAO/WHO equations (Biolo et al., 2008; Bosutti et al., 2008; Debevec et al., 2014). Dietary intake was controlled to maintain eucaloric condition (Biolo et al., 2008; Bosutti et al., 2008; Debevec et al., 2014). A metabolic test to determine glutathione kinetics was performed at the end of each intervention period (i.e., normoxic bed rest, hypoxic bed rest and hypoxic ambulatory) and at the end of one of the three pre-intervention periods (i.e., normoxic ambulatory) (Biolo et al., 2008). At the same time, blood samples were taken to measure inflammatory, metabolic and redox biomarkers. Body composition was determined by DXA (Debevec et al., 2014).

In the morning, after an overnight fast, the metabolic test was started approximately at 07.00 AM. In each volunteer, two polyethylene catheters were inserted: one into a forearm vein, for isotope infusion, the other into a wrist vein of the opposite arm, heated at 50 °C, to obtain arterialized venous blood. After background blood sample collection, a 6-h primed-continuous infusions of ^2^H_2_-glycine (26.5 μmol × kg-1 × h-1), (Cambridge Isotope Laboratories, Andover, MA, United States) was started. Background blood samples were collected to assess in erythrocytes natural isotopic enrichments for ^2^H_2_-glycine and ^2^H_2_-glutathione as well as GCLc and GCLm expression, total glutathione concentration, GSH/GSSG, cysteine, glutamate, glutamine, glycine and membrane fatty acid composition (Biolo et al., 2008, 2018; Agostini et al., 2010; Mazzucco et al., 2010a). The same background blood samples were used to measure: plasma levels of insulin, glucose, total and HDL-C, HDL2-C and HDL3-C sub-fractions, triglycerides, FFA, LCAT, CETP, HL, LPL, CRP, SAA, TRAIL, presepsin, homocysteine, pyroglutamic acid, glutamate, cysteine, glutamine, and glycine (Pišot et al., 1985; Mazzucco et al., 2010b; Biolo et al., 2012; Qi et al., 2013). During the metabolic test, blood samples were drawn at 180, 270, and 360 min to assess enrichments of ^2^H_2_-glycine and [^2^H_2_-glycine]-glutathione in red blood cells. Total glutathione concentrations were determined in erythrocytes also at the end of tracer infusions to assess metabolic steady state during the tracer incorporation period. All blood samples were collected in EDTA tubes and immediately centrifuged at 3000 *g* at 4°C for 10 min; plasma and erythrocytes were separated and stored at -80°C. The hematocrit was assessed at the end of each metabolic test.

### Analyses

Isotopic enrichments of glycine and glutathione in erythrocytes, were determined by GC-MS (HP 5890; Agilent Technologies, Santa Clara, CA, United States) as previously described (Biolo et al., 2008; Agostini et al., 2010). Isotopic enrichments were assessed considering the following mass-to-charge ratios (m × z-1): glycine m × z-1 218-220; glutathione m × z-1 363-366. Erythrocyte total glutathione concentrations were determined using the internal standard approach, through the addiction of known amount of [^13^C_2_-^15^N-glycine]-glutathione (Cambridge Isotope Laboratories, Andover, MA, United States). Molecular expression of GCLc and GCLm, the two subunits of the key enzyme for glutathione synthesis, were determined by Western blot analysis in erythrocytes and expressed as fraction of GAPDH (Biolo et al., 2008). Amino acid concentrations in plasma and erythrocytes were assessed by GC-MS using the internal standard technique (Biolo et al., 2008), monitoring the following m × z^-1^: cysteine m × z^-1^ 406/408, glutamate m × z^-1^ 432/433, glutamine m × z^-1^ 431/432, glycine m × z^-1^ 218/219. Plasma homocysteine levels were assessed at wm × z^-1^ 496/500. Pyroglutamic acid was measured in plasma as ratio between pyroglutamic acid and glutamate area, detected at the m × z^-1^ 300 and 432, respectively (Qi et al., 2013). Plasma insulin, glucose, high-sensitivity CRP, total cholesterol, HDL-C and triglycerides were measured with standard methods by a certified external laboratory (Synlab Italia Srl, Italy). Commercially available kits were used to determine FFA plasma concentrations (Cayman Chemical Company, Ann Arbor, MI, United States), CETP (ALPCO, Salem, NH, United States), LCAT (MyBioSource, San Diego, CA, United States), LPL (MyBioSource, San Diego, CA, United States), HL (MyBioSource, San Diego, CA, United States), SAA (SAA, Abcam, United Kingdom), TRAIL (R&D Systems, Minneapolis, MN, United States) as well as the GSH/GSSG in erythrocytes (Oxford Biomedical Research Inc., Rochester Hill, MI, United States). HDL2 and HDL3 cholesterol concentrations were assessed by the precipitation technique (Gesan Production srl, Italy and Fitzgerald Industries International, Acton, MA, United States). Erythrocyte membrane fatty acid composition was assessed as % of total content by gas-chromatography-flame ionization detection (GC-FID; GC 6850 Agilent Technologies, Santa Clara, CA, United States), as previously reported (Mazzucco et al., 2010a).

### Calculations

Glutathione FSR, % × d^-1^, was calculated according to the precursor ([^2^H_2_]-glycine) into product ([^2^H_2_]-glutathione) incorporation approach (Agostini et al., 2010; Biolo et al., 2018):

(E[^2^H_2_]-glutathione × t^-1^) × E[^2^H_2_]-glycine^-1^× 24h × 100 where (E[^2^H_2_]-glutathione × t^-1^) is the slope of the regression line describing the rise in erythrocyte [^2^H_2_]-glutathione enrichment as a function of time (hours) and E[^2^H_2_]-glycine is the mean glycine enrichment in erythrocytes at steady state. The ASR was calculated as the product of FSR and glutathione concentrations. Glutathione concentrations and ASR were normalized by erythrocyte volume or by whole blood volume, according to the hematocrit.

Plasma LDL-C was calculated by the Friedewald’s formula (Voltan et al., 2016). The delta-5 desaturase activity index was calculated as ratio between membrane content of arachidonic acid (20:4 n-6) and dihomo-γ-linoleic acid (20:3 n-6) (Liu et al., 2015). The n-3 index was calculated as sum of membrane docosahexaenoic (22:6, n-3) (DHA) and eicosapentaenoic acid (20:5, n-3) (EPA) (Liu et al., 2015). The Homeostasis Model Assessment of insulin resistance (HOMA2-IR) score was calculated with the use of HOMA Calculator, version 2.2.3 (The University of Oxford 2013) (Levy et al., 1998).

### Statistics

All data are expressed as mean ± SEM. Results in the 4 different experimental conditions (normoxia and hypoxia in ambulatory condition, normoxia and hypoxia in bed rest) were analyzed by using a repeated-measures ANOVA or co-variance (ANCOVA) with activity (ambulatory or bed rest) and oxygen level (normoxia and hypoxia) as the two factors. If interactions were significant, *post hoc* analysis was performed using Student *t*-test with Bonferroni correction. *P*-value < 0.05 was considered statistically significant. Data were log transformed when appropriate. Hypoxia–mediated changes from the normoxic state, in the ambulatory and bed rest conditions, were compared by using Student’s paired *t*-test. The Pearson’s correlation coefficient was used to measure the strength of linear associations between hypoxia–mediated changes of different variables from the normoxic state in the ambulatory and bed rest conditions (*n* = 22). Statistical analysis was performed using SPSS statistical software (version 21; SPSS, Inc., Chicago, IL, United States).

## Results

### Insulin, Inflammatory Markers and TRAIL

**FIGURE 1:**
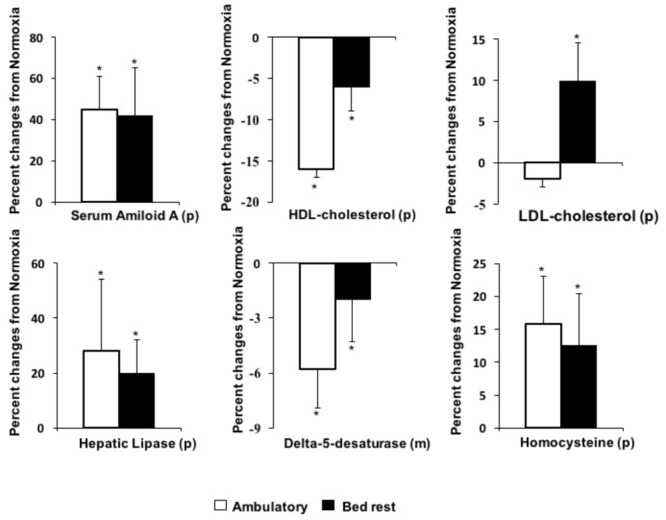
Effects of hypoxia in ambulatory or bed rest conditions on pro-atherogenic biomarkers. (p) determined in plasma or serum; (m) determined in erythrocyte membrane; ^∗^*P* < 0.05, indicates significant effects of hypoxia based on repeated measures ANOVA or ANCOVA. If interactions were significant, *post hoc* analysis was performed using Student *t*-test with Bonferroni correction.

Results of changes in fat mass and lean body mass, as assessed by DXA, are reported elsewhere (Debevec et al., 2014). Lean body mass significantly decreased in all the three conditions (i.e., hypoxia ambulatory, normoxia bed rest, hypoxia bed rest) by about 4%, while fat mass did not change significantly. There were not significant effects of hypoxia or bed rest on plasma glucose and insulin concentrations in the postabsorptive state as well as on the HOMA2-IR score (**Table 1**). Hypoxia, but not bed rest exposure, significantly increased inflammatory markers, i.e., CRP and SAA concentrations (**Figure 1**). There was not significant bed rest × hypoxia interaction on inflammatory markers (**Table 1**). Plasma TRAIL concentration was significantly increased by hypoxia and a significant interaction between physical activity and oxygen levels was observed (**Figure 1**). Hypoxia mediated increases of TRAIL concentrations were greater (*p* = 0.01) in ambulatory conditions (+34 ± 6%) than during bed rest (+17 ± 7%). There was a significant interaction between physical activity and oxygen level for plasma concentrations of presepsin, biomarker of innate immunity. Bed rest significantly increased presepsin level in hypoxic conditions, while this effect was blunted in normoxia. Bed rest mediated changes of presepsin concentrations were greater (*p* = 0.03) in hypoxic conditions (+36 ± 14%) than in normoxia (+9 ± 16%).

**Table 1:** Effects of hypoxia and bed rest on circulating insulin, inflammatory mediators, and TRAIL^1^.

	Ambulatory		Bed rest		*P*		
	Normoxia	Hypoxia	Normoxia	Hypoxia	Bed rest effect	Hypoxia effect	Interaction
Insulin (μU⋅mL^-1^)	7 ± 1	7 ± 1	7 ± 1	6 ± 1	0.85	0.93	0.27
Glucose (mg⋅dL^-1^)	94 ± 2	93 ± 1	93 ± 1	92 ± 1	0.35	0.57	1.00
HOMA2-IR	0.69 ± 0.01	0.68 ± 0.01	0.68 ± 0.01	0.67 ± 0.01	0.35	0.56	1.00
Homocysteine (μmol × L^-1^)	15.3 ± 1.7	17.0 ± 1.4	14.7 ± 1.4	16.2 ± 1.4	0.38	0.003	0.93
C-reactive protein (mg⋅L^-1^)	0.6 ± 0.1	1.7 ± 1.0	0.4 ± 0.1	0.8 ± 0.2	0.38	0.04	0.82
Serum amyloid A (μg⋅L^-1^)	19.6 ± 0.7	28.3 ± 3.0	22.9 ± 2.4	28.9 ± 3.6	0.26	0.03	0.59
Presepsin (pg/ml)	91 ± 8	70 ± 6*	101 ± 13	107 ± 11	0.13	0.18	0.01
TRAIL (pg/ml)	80 ± 4	107 ± 7*	82 ± 4	94 ± 5	0.21	0.001	0.01

*HOMA2-IR, Homeostatic Model Assessment of Insulin resistance score; TRAIL, TNF-related apoptosis-inducing ligand. Data are expressed as mean ± SEM. Data were analyzed with the use of a 2-factor repeated measure ANOVA or ANCOVA. *Post hoc* analysis was performed using Student *t*-test with Bonferroni correction. ^∗^*p* < 0.05 hypoxia versus normoxia.*

### Circulating Lipids

**FIGURE 2:**
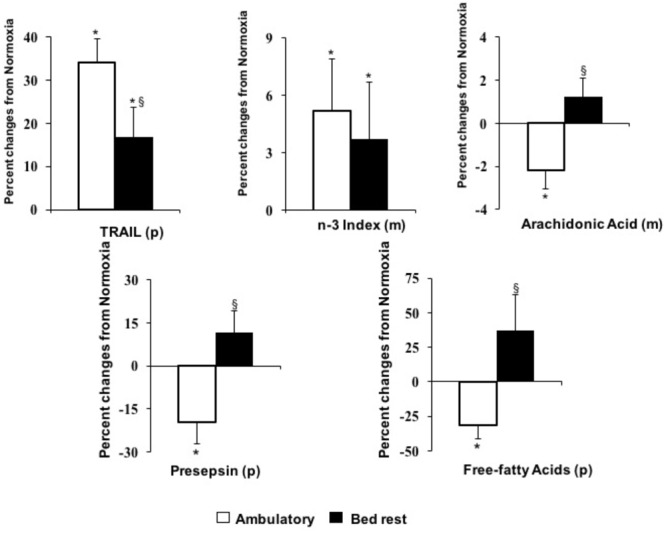
Effects of hypoxia in ambulatory or bed rest conditions on anti-atherogenic biomarkers. (p) determined in plasma or serum; (m) determined in erythrocyte membrane. ^∗^*P* < 0.05, indicates significant effects of hypoxia based on repeated measures ANOVA or ANCOVA. If interactions were significant, *post hoc* analysis was performed using Student t-test with Bonferroni correction. ^§^ *P* < 0.05, indicates significantly different from ambulatory based on Student *t*-test for paired data.

Effects of hypoxia *per se* and/or combined to bed rest on plasma lipid concentrations as well as on key enzymes involved in lipid metabolism are reported in **Table 2**. Plasma levels of total cholesterol were not significantly affected by 10-day bed rest, *per se* or associated to hypoxia. There was a significant interaction between physical activity and oxygen level for plasma concentrations of LDL-C (**Figure 1**). Hypoxia significantly increased LDL-C in bed rest by 10 ± 5%. This effect was blunted in ambulatory conditions. Bed rest and hypoxia additively decreased HDL-C (**Figure 1**): the first significantly lowering HDL3-C and the second HDL2-C sub-fraction concentrations. The ratio between HDL2-C and HDL3-C significantly decreased following bed rest. There was a significant bed rest × hypoxia interaction on the ratio between HDL2-C and HDL3-C. Bed rest-induced decline of the ratio between HDL2-C and HDL3-C was significantly (*p* = 0.05, Wicoxon test) greater in hypoxia (-0.39 ± 0.28) than in normoxia (-0.20 ± 0.32). The ratio between total and HDL-C concentrations were significantly decreased both by bed rest and hypoxia. Hypoxia had a significant positive effect on triglyceride concentrations, with a significant interaction between physical activity and oxygen levels for plasma concentrations of FFA (**Figure 2**). Hypoxia in ambulatory conditions significantly decreased FFA concentrations, while these effects were blunted with bed rest. Hypoxia mediated changes in FFA concentrations were lower (*p* = 0.03) in ambulatory conditions (-31 ± 10%) than during bed rest (+37 ± 26%). Bed rest and/or hypoxia had different effects on enzymes involved in lipid metabolism. Neither short-term physical inactivity and/or hypoxia affected circulating levels of LCAT and CEPT. Exposure to low oxygen pressure significantly decreased LPL and increased HL levels (**Figure 1**).

**Table 2:** Effects of hypoxia and bed rest on circulating lipids and enzyme involved in lipid metabolism.

	Ambulatory		Bed rest		*P*		
	Normoxia	Hypoxia	Normoxia	Hypoxia	Bed rest effect	Hypoxia effect	Interaction
**Plasma lipids**							
Total cholesterol (mg⋅dL^-1^)	184 ± 13	173 ± 8	174 ± 10	180 ± 11	0.66	0.67	0.12
LDL-C (mg⋅dL^-1^)	117 ± 12	110 ± 7	110 ± 7	121 ± 9*	0.17	0.18	0.01
HDL-C (mg⋅dL^-1^)	46 ± 10	33 ± 2	32 ± 2	28 ± 2	<0.01	0.02	0.65
HDL2-C (mg⋅dL^-1^)	19 ± 9	9 ± 1	10 ± 1	7 ± 1	0.17	0.04	0.44
HDL3-C (mg⋅dL^-1^)	26 ± 2	24 ± 2	22 ± 2	21 ± 2	0.01	0.27	0.62
HDL2-C/HDL3-C	0.72 ± 0.28	0.41 ± 0.8	0.51 ± 0.09	0.33 ± 0.03	0.03	0.19	0.001
Total/HDL cholesterol	4.0 ± 0.5	4.4 ± 0.4	4.3 ± 0.3	4.8 ± 0.4	<0.001	<0.01	0.78
Triglycerides (mg⋅dL^-1^)	94 ± 14	113 ± 14	109 ± 20	102 ± 13	0.40	0.09	0.61
Free fatty acid (μmol/L)	286 ± 21	193 ± 28*	205 ± 25	239 ± 26	0.35	0.17	0.02
**Enzymes involved in lipid metabolism**							
Cholesteryl ester transfer protein (μg⋅mL^-1^)	2.81 ± 0.18	2.93 ± 0.18	2.96 ± 0.25	3.09 ± 0.16	0.32	0.31	0.95
Lecithin-cholesterol acyltransferase (ng⋅L^-1^)	21.8 ± 4.2	20.3 ± 4.3	21.7 ± 4.7	23.8 ± 6.3	0.66	0.99	0.45
Lipoprotein lipase (ng⋅L^-1^)	31.1 ± 3.3	24.5 ± 3.7	30.5 ± 4.8	25.8 ± 3.7	0.82	0.02	0.54
Hepatic lipase (U⋅mL^-1^)	0.72 ± 0.10	0.79 ± 0.09	0.80 ± 0.09	0.90 ± 0.08	0.15	0.04	0.81
LPL-to-HL ratio	56 ± 13	33 ± 5	39 ± 4	30 ± 5	0.15	0.03	0.31

*Data are expressed as mean ± SEM. Data were analyzed with the use of a 2-factor repeated measure ANOVA or ANCOVA. *Post hoc* analysis was performed using Student *t*-test with Bonferroni correction. ^∗^*p* < 0.05 hypoxia versus normoxia.*

### Cell Membrane Lipid Composition

Results of the effects of hypoxia and bed rest on lipid composition in red blood cell membranes are reported in **Table 3**. There was no effect of hypoxia or bed rest on membrane content of saturated (myristic, palmitic and stearic) or monounsaturated (palmitoleic, oleic, trans elaidic and eicosenoic) fatty acids. Among the PUFA, hypoxia and bed rest significantly increased membrane content of DHA (22:6, n-3), while there were no changes in EPA (20:5, n-3) levels. The sum of these n-3 PUFA defined as n-3 index was significantly increased by hypoxia (**Figure 2**). Membrane content of arachidonic acid (20:4n-6) was increased by bed rest, while hypoxia decreased this pro-inflammatory PUFA in ambulatory conditions but not in bed rest. The delta-5 desaturase index, derived from the ratio between arachidonic and dihomo-γ-linolenic acids, was significantly reduced by exposure to both bed rest and hypoxia (**Figure 1**). This index of insulin sensitivity decreased by 5 ± 2% after 10 days of physical inactivity and by 6 ± 2% after 10 days of hypoxia, in comparison to baseline conditions. No interaction was observed between bed rest and hypoxia on the delta-5 desaturase index.

**Table 3:** Effects of hypoxia and bed rest on erythrocyte membranes fatty acid composition.

	Ambulatory		Bed rest		*P*		
	Normoxia	Hypoxia	Normoxia	Hypoxia	Bed rest effect	Hypoxia effect	Interaction
Myristic 14:00	0.20 ± 0.02	0.31 ± 0.32	0.30 ± 0.03	0.25 ± 0.02	0.45	0.08	0.01
Palmitic 16:00	20.6 ± 0.4	20.9 ± 0.3	20.6 ± 0.3	20.8 ± 0.3	0.71	0.15	0.76
Stearic 18:00	18.5 ± 0.2	18.4 ± 0.1	18.6 ± 0.2	18.5 ± 0.1	0.58	0.27	9.64
Palmitoleic 16:1 n7	0.25 ± 0.04	0.27 ± 0.04	0.28 ± 0.04	0.26 ± 0.03	0.72	1.00	0.26
Oleic 18:1 n9	13.3 ± 0.3	12.8 ± 0.3	12.8 ± 0.3	12.9 ± 0.3	0.08	0.10	0.06
Elaidic trans 18:1n-9	1.03 ± 0.04	1.02 ± 0.03	1.01 ± 0.05	1.05 ± 0.05	0.70	0.45	0.14
Eicosenoic 20:1n-9	0.31 ± 0.01	0.3 ± 0.01	0.3 ± 0.01	0.31 ± 0.01	0.86	0.75	0.10
Eicosapentaenoic 20:5 n-3	0.53 ± 0.04	0.58 ± 0.04	0.56 ± 0.03	0.54 ± 0.04	0.94	0.50	0.10
Docosapentaenoic 22:5 n-3	2.50 ± 0.08	2.57 ± 0.10	2.55 ± 0.09	2.57 ± 0.09	0.43	0.02	0.41
Docosahexaenoic 22:6 n-3	4.48 ± 0.23	4.69 ± 0.26	4.63 ± 0.25	4.80 ± 0.2	0.02	0.01	0.83
Linoleic 18:2 n6	91.9 ± 0.35	11.7 ± 0.3	11.6 ± 0.35	11.39 ± 0.29	0.08	0.21	0.83
Eicosadienoic 20:2 n-6	1.09 ± 0.51	1.47 ± 0.56	1.13 ± 0.19	0.89 ± 0.24	0.60	0.63	0.50
Dihomo-γ-linolenic 20:3 n-6	1.97 ± 0.13	2.04 ± 0.13	2.08 ± 0.12	2.16 ± 0.13	0.001	0.02	0.98
Arachidonic 20:4 n-6	18.1 ± 0.3	17.7 ± 0.3*	18.2 ± 0.2	18.4 ± 0.3	0.04	0.49	0.02
Adrenic 22:4 n-6	4.15 ± 0.20	3.99 ± 0.18	4.12 ± 0.17	4.17 ± 0.18	0.07	0.02	0.02
Docosapentaenoic 22:5 n-6	0.99 ± 0.08	1.10 ± 0.13	1.17 ± 0.17	0.98 ± 0.08	0.71	0.47	0.17
n-3 index^1^	5.01 ± 0.25	5.27 ± 0.29	5.19 ± 0.26	5.34 ± 0.20	0.06	0.02	0.58
Delta-5 desaturase^2^	9.6 ± 0.6	9.0 ± 0.5	9.1 ± 0.6	8.9 ± 0.6	0.03	0.02	0.15

*Values are percent of total membrane fatty acids (mean ± SEM). ^*1*^n-3 index, sum of eicosapentaenoic and docosahexaenoic acids. ^*2*^Delta-5 desaturase, ratio between arachidonic and dihomo-g-linolenic acids. Data were analyzed with the use of a 2-factor repeated measure ANOVA or ANCOVA. *Post hoc* analysis was performed using Student *t*-test with Bonferroni correction. ^∗^*p* < 0.05 hypoxia versus normoxia.*

### Glutathione Redox Capacity

**FIGURE 3:**
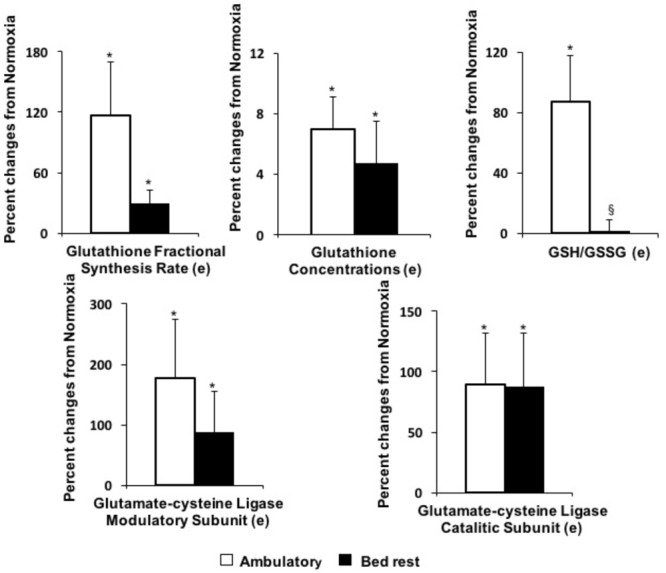
Effects of hypoxia in ambulatory or bed rest conditions on erythrocyte glutathione status. (e) Determined in erythrocyte cytoplasm. ^∗^*P* < 0.05, indicates significant effects of hypoxia based on repeated measures ANOVA or ANCOVA. If interactions were significant, *post hoc* analysis was performed using Student *t*-test with Bonferroni correction. ^§^ *P* < 0.05, indicates significantly different from ambulatory based on Student *t*-test for paired data.

**Table 4** shows the effects of hypoxia and bed rest *per se*, or in combination, on glutathione status in erythrocytes. Hypoxia had a significant effect on the hematocrit, thus results about glutathione concentrations and ASR were normalized by both erythrocyte and whole blood volume. Total glutathione concentrations in erythrocytes, normalized by whole blood volume, significantly increased following hypoxia leading to increased availability of total circulating glutathione. There was not significant bed rest × hypoxia interaction. The GSH/GSSG ratio is an index of redox balance. Hypoxia × bed rest interaction on GSH/GSSG was significant. Hypoxia significantly increased redox glutathione capacity (i.e., GSH/GSSG) in ambulatory conditions (+87 ± 31%), while this effect was blunted in bed rest. There was significant hypoxia × bed rest interaction on the oxidized form of glutathione (GSSG), normalized by both erythrocyte and whole blood volumes. GSSG (normalized by erythrocyte volume) was significantly decreased (-28 ± 14%) by hypoxia in ambulatory conditions, but this effect was blunted in bed rest. There were positive effects of hypoxia in ambulatory and bed rest conditions on the FSR and ASR of glutathione in erythrocytes, as determined by deuterated-glycine incorporation into the tripeptide. The western blot protein expression in erythrocytes of the catalytic (GCL-C) and modulatory (GCL-M) subunits of glutamate-cysteine ligase, the key enzyme for glutathione synthesis, was significantly increased by hypoxia (**Figure 3**).

**Table 4:** Effects of hypoxia and bed rest on glutathione redox capacity in erythrocytes (RBC) and in whole blood (WB).

	Ambulatory		Bed rest		*P*		
	Normoxia	Hypoxia	Normoxia	Hypoxia	Bed rest effect	Hypoxia effect	Interaction
Hematocrit (percent)	46 ± 1	51 ± 1	47 ± 1	51 ± 1	0.20	<0.001	0.12
Total glutathione (μmol/L RBC)	2692 ± 84	2620 ± 79	2617 ± 105	2547 ± 86	0.07	0.21	0.34
Total glutathione (μmol/L WB)	1245 ± 49	1331 ± 52	1236 ± 54	1287 ± 51	0.22	0.01	0.45
GSH/GSSG^1^ (ratio)	137 ± 26	213 ± 34*	207 ± 41	176 ± 36	0.93	0.52	0.02
GSSG (μmol/L RBC)	32 ± 9	19 ± 5*	21 ± 5	38 ± 20	0.87	0.43	0.02
GSSG (μmol/L WB)	15 ± 4	9 ± 2	10 ± 2	19 ± 10	0.89	0.76	0.02
Glutathione FSR^2^ (percent/day)	25 ± 4	38 ± 6	35 ± 3	41 ± 3	0.09	0.03	0.21
Glutathione ASR^3^ (μmol/day/L RBC)	669 ± 122	990 ± 143	898 ± 82	1053 ± 88	0.10	0.02	0.19
Glutathione ASR (μmol/day/L WB)	306 ± 55	493 ± 69	420 ± 40	524 ± 41	0.23	0.02	0.27
GCL-C^4^ (fraction of GAPDH^5^)	0.63 ± 0.09	1.01 ± 0.25	0.64 ± 0.10	0.85 ± 0.14	0.96	0.03	0.91
GCL-M^6^ (fraction of GAPDH)	0.82 ± 0.15	1.29 ± 0.14	0.87 ± 0.13	1.05 ± 0.12	0.74	0.05	0.27

*Data are expressed as mean ± SEM. Data were analyzed with the use of a 2-factor repeated measure ANOVA or ANCOVA. *Post hoc* analysis was performed using Student *t*-test with Bonferroni correction. ^∗^*p* < 0.05 hypoxia versus normoxia. ^*1*^GSH/GSSG, ratio between reduced and oxidised glutathione. ^*2*^FSR, fractional synthesis rate; ^*3*^ASR, absolute synthesis rate; ^*4*^GCL-C, glutamate cysteine ligase– catalytic subunit; ^*5*^GAPDH, glyceraldehyde-3-phosphate dehydrogenase; ^*6*^GCL-M, glutamate cysteine ligase–modulator subunit.*

### Selected Amino Acids

Concentrations of selected amino acids in plasma and erythrocyte cytoplasm are reported in **Table 5**. Hypoxia significantly increased plasma homocysteine concentrations in both bed rest and ambulatory conditions (**Figure 1**). There was significant positive effect of hypoxia on plasma concentrations of the glutathione precursors, cysteine and glutamate. Plasma glycine, the third glutathione precursor, was increased by hypoxia only in ambulatory conditions. The ratio between plasma pyroglutamic acid and glutamate, an index inversely associated with the rate of glutathione re-synthesis through the γ-glutamyl cycle, was decreased by hypoxia, in both bed rest and ambulatory conditions. Hypoxia had a significant negative effect of on plasma glutamine concentrations.

**Table 5:** Effects of hypoxia and bed rest on selected amino acid concentrations in plasma and red blood cells.

	Ambulatory		Bed rest		*P*		
	Normoxia	Hypoxia	Normoxia	Normoxia	Bed rest effect	Hypoxia effect	Interaction
**Plasma amino acid concentrations**			
Homocysteine (μmol × L^-1^)	15.3 ± 1.7	17.0 ± 1.4	14.7 ± 1.4	16.2 ± 1.4	0.38	0.003	0.93
Cysteine (μmol × L^-1^)	294 ± 12	299 ± 11	277 ± 10	304 ± 11	0.45	0.005	0.27
Glycine (μmol × L^-1^)	172 ± 9	185 ± 10*	196 ± 12	185 ± 12	0.04	0.78	0.01
Glutamate (μmol × L^-1^)	61 ± 4	69 ± 5	62 ± 3	67 ± 3	0.81	0.03	0.59
Glutamine (μmol × L^-1^)	541 ± 10	515 ± 9	552 ± 21	522 ± 7	0.55	0.01	0.86
Pyroglutamic acid^a^ (μmol × L^-1^)	1.02 ± 0.08	0.90 ± 0.06	1.01 ± 0.07	0.89 ± 0.06	0.85	0.04	0.86
**Erythrocyte amino acid concentrations**			
Cysteine (μmol × L^-1^)	70 ± 3	79 ± 3	72 ± 4	70 ± 3	0.20	0.33	0.07
Glycine (μmol × L^-1^)	368 ± 11	389 ± 13	372 ± 12	393 ± 12	0.49	<0.001	0.98
Glutamate (μmol × L^-1^)	477 ± 24	481 ± 18	448 ± 16	478 ± 17	0.33	0.16	0.37
Glutamine (μmol × L^-1^)	1053 ± 147	1015 ± 209	1042 ± 141	1000 ± 215	0.79	0.73	0.97

*Data are expressed as mean ± SEM. Data were analyzed with the use of a 2-factor repeated measure ANOVA or ANCOVA. *Post hoc* analysis was performed using Student t-test with Bonferroni correction. ^∗^*p* < 0.05 hypoxia versus normoxia.*

## Discussion

We have investigated the effects of 10-days of hypoxia, associated with bed rest or moderate level of standardized physical activity, on development of pro- and anti-atherogenic factors in healthy volunteers. Hypoxia, either in bed rest or ambulatory condition: (a) activated systemic inflammation; (b) lowered plasma levels of total HDL-C, HDL2-C sub-fraction and decreases HL; (c) increased plasma homocysteine; (d) decreased delta-5 desaturase index in RBC membrane. The combination of bed rest and hypoxia additively decreased total HDL-C and delta-5 desaturase index. In parallel to these pro-atherogenic effects, hypoxia activated selected anti-atherogenic pathways including anti-inflammatory (circulating TRAIL and membrane omega-3 index) and antioxidant (higher synthesis rate of intracellular glutathione) mechanisms. When hypoxia was applied in condition of moderate physical activity, antioxidant glutathione capacity (i.e., GSH/GSSG) increased, TRAIL was up-regulated, while circulating FFA and cell membrane arachidonic acid decreased. Hypoxia during bed rest blunted these anti-atherogenic effects and increases circulating LDL-C. These results clearly show that following 10-day hypoxia a number of pro- and anti-atherogenic pathways are activated. Nonetheless, a moderate level of physical activity in hypoxia enhances anti-atherogenic pathways and blunts cardiometabolic risk.

### Pro-atherogenic Biomarkers

Experimental hypoxia, either in ambulatory or bed rest conditions, was associated with sub-clinical activation of the systemic inflammatory response in healthy young volunteers, as shown by increments in circulating levels of the acute phase proteins CRP and SAA. This hypoxia-mediated activation of inflammatory mediators is in agreement with previous studies (Eltzschig and Carmeliet, 2011). In the clinical setting, hypoxia may contribute to the systemic inflammation associated with a number of diseases (i.e. COPD, OSAS and heart failure) (Eltzschig and Carmeliet, 2011), most likely by a direct activation of NF-kB or through HIF-1 (Eltzschig and Carmeliet, 2011; Bruning et al., 2012). Inflammation, as part of the innate immunity, contributes to atherosclerosis (Eltzschig and Carmeliet, 2011; Sasaki et al., 2012). Presepsin is a new biomarker of innate immunity (Chenevier-Gobeaux et al., 2015), which has been proposed for early diagnosis of bacterial infection and ST elevation myocardial infarction (Caglar et al., 2017). For the first time we have shown that hypoxia has the potential to activate presepsin. This effect has been demonstrated in bed rest but not in ambulatory conditions.

As shown, 10 days of bed rest during hypoxia additively decreased HDL-C. Based on physicochemical properties, HDL are categorized into two major sub-fractions: HDL2, larger and more buoyant and HDL3, smaller and denser. Our results indicate that hypoxia preferentially decreases the large HDL2, while bed rest had significant negative effects on the small and dense HDL3. In agreement with our findings, previous studies indicate that short-term bed rest preferentially decreases HDL3 sub-fraction (Yanagibori et al., 1998), while lung inflammatory diseases are associated with decreased HDL2 (Salazar et al., 1998; Kim et al., 2016). Clinical studies suggest that both HDL2-C and HDL3-C sub-fractions are inversely associated with the incidence of coronary artery disease (Camont et al., 2011). In our study, the HDL2-C-to-HDL3-C ratio decreased following bed rest, especially in hypoxic condition. The same pattern is observed also in conditions of increased cardiometabolic risk such as insulin resistance, menopause and systemic inflammation (Moriyama and Takahashi, 2016).

As for circulating levels of enzymes involved in lipid metabolism, neither short-term physical inactivity nor hypoxia, isolated or combined, affected CETP and LCAT concentrations. We have previously shown that 35-days of bed rest significantly increase circulating CETP (Mazzucco et al., 2010b). In the present study, CETP tended to increase without achieving statistical significance. In contrast to LCAT and CETP, exposure to low oxygen pressure significantly increased circulating HL and decreased LPL. HL is a glycoprotein responsible for faster HDL clearance. This enzyme transforms HDL2 to HDL3 particles, which are removed quickly from the bloodstream by the liver (Chatterjee and Sparks, 2011). LPL hydrolyzes triglycerides in chylomicrons and VLDL. Reduction of LPL activity may decrease triglyceride clearance and increase triglyceride-rich lipoproteins. This mechanism can allow the CETP-mediated exchange of triglycerides from ApoB triglyceride-rich lipoproteins (ApoB-TRL) to HDL, and of cholesteryl-esters from HDL to ApoB-TRL. Such modifications in HDL composition may lead to increased HL activity and HDL clearance (Chatterjee and Sparks, 2011). Systemic inflammation can also contribute to the observed hypoxia-mediated HDL reduction. SAA may be transiently associated with HDL decline during acute inflammation. This acute-phase protein may displace ApoA-I, leading to increased catabolism of HDL (McEneny et al., 2016). Our results show that following hypoxia, lipoprotein pattern shifts toward a more atherogenic lipid profile by lowering total HDL-C and HDL2-C sub-fraction. LDL-C can also be affected by the interaction between hypoxia and bed rest. Hypoxia increased LDL-C in bed rest, but not in ambulatory conditions. Potential effects of hypoxia, bed rest and physical activity on LDL-C have been previously shown (Nakano et al., 2005; Yang et al., 2014). These results further suggest that hypoxia associated to moderate level of physical activity may decrease cardiometabolic risk.

The effects of bed rest and normobaric hypoxia on insulin resistance have been shown elsewhere (Simpson et al., 1985). In this study, bed rest and hypoxia additively decrease the delta-5 desaturase index, a sensitive and reliable marker of insulin sensitivity (Pan et al., 1995; Mazzucco et al., 2010a). The reduction of the activity of this enzyme can alter insulin response by lowering PUFA within cell membranes (Pan et al., 1995; Mazzucco et al., 2010a). Insulin resistance may further decrease HDL plasma levels (Mazzucco et al., 2010b). Decreased delta-5 desaturase index has been associated with atherosclerosis and cardiovascular diseases (Lu et al., 2012; Daneshmand et al., 2017).

Plasma homocysteine level is a strong mortality predictor in patients with coronary artery disease (Nygård et al., 1997). Hypoxia increased circulating homocysteine, by about 10%, in both ambulatory and bed rest condition. Such an evidence matches *in vitro* studies indicating hypoxia as an inhibitor of the homocysteine re-methylation pathway, with consequent accumulation of this metabolite (Avila et al., 1998).

Glutamine is the most abundant amino acid in blood and tissue cells. It is required by the immune system both as a primary fuel and as a carbon and nitrogen donor for nucleotide synthesis (Biolo et al., 2005b). In our study, hypoxia decreased plasma glutamine by about 4%. Decreased circulating glutamine is associated with systemic inflammation (Biolo et al., 2005b) and accelerated atherosclerosis (Hasokawa et al., 2012).

### Anti-atherogenic Biomarkers

Convincing evidence indicates that circulating levels of TRAIL are inversely related to the risk of cardiovascular mortality (Volpato et al., 2011) and, in experimental conditions, exogenous TRAIL administration exhibited anti-atherosclerotic activity (Forde et al., 2016). In human experimental bed rest, there is a positive direct relationship between energy balance and TRAIL levels (Biolo et al., 2012). Physical inactivity tended to downregulate TRAIL without achieving statistical significance (Biolo et al., 2012). In the present study, in agreement with previous observations in animals (Forde et al., 2016), hypoxia directly increases circulating TRAIL and, this effect is twice greater in ambulatory conditions than in bed rest. This data suggests that a moderate level of physical activity, in hypoxic conditions, can stimulate circulating level of TRAIL.

We have previously shown that long-term bed rest is associated with increased membrane content of the pro-inflammatory arachidonic acid (Mazzucco et al., 2010a), which can directly promote atherosclerosis (Harris et al., 2017). In the present study, the increase of arachidonic acid is confirmed also after a short-term bed rest in normoxia. In contrast, hypoxia in ambulatory condition significantly reduced arachidonic acid levels. However, this potential anti-atherogenic effect was blunted during hypoxic bed rest. At the same time, in agreement with a previous study in rats (Jezková et al., 2002), we found that hypoxia increased n3-PUFA content in erythrocyte membranes leading to 30% increase in the n3 index, in both bed rest and ambulatory conditions. A higher n3-index is associated with reduced cardiovascular mortality independently of other risk factors (Watkins and Hotamisligil, 2012).

Glutathione is a critical factor in protecting cells against oxidative stress. Glutathione redox capacity depends on the availability of total glutathione and by the GSH/GSSG ratio. We have observed that hypoxia directly increased total whole blood glutathione concentration. Such increase in total glutathione availability was paralleled by acceleration in the rate of glutathione synthesis expressed either as FSR or ASR. In agreement with the kinetic results, we found that hypoxia directly increased both the modulatory and catalytic subunits of glutamate cysteine ligase, the key enzyme for glutathione synthesis, as determined by western blot in erythrocytes. Glutathione is catabolized and re-synthetized through the gamma-glutamyl cycle. Pyroglutamic acid is the product of glutathione catabolism and the immediate precursor of glutamate available for glutathione re-synthesis. The ratio between pyroglutamic acid and glutamate is therefore an index of systemic glutathione turnover (Qi et al., 2013). We found that the ratio between plasma levels of pyroglutamic acid and glutamate decreased in hypoxic conditions. This result suggests an increased activity of the gamma-glutamyl cycle and of glutathione turnover, not only in erythrocytes but also at the whole body level. The hypoxia-mediated increase in glutathione turnover was paralleled by an increment in plasma concentrations of all precursor amino acids, i.e., glycine, glutamate and cysteine. In addition to the effect on total glutathione availability, hypoxia in ambulatory conditions also improved the GSH/GSSG ratio suggesting a decreased production of reactive oxygen species. In contrast to the ambulatory condition, hypoxia in bed rest did not change significantly the GSH/GSSG ratio. Erythrocyte GSSG levels increased by about 100% following hypoxia in bed rest conditions, while it tended to decrease in ambulatory conditions. Hypoxia is known to elicit production of reactive oxygen species and activate antioxidant systems, as intracellular glutathione. The redox balance is also influenced by the level of physical activity. Both strenuous exercise and muscle unloading have been shown to promote oxidative stress. In agreement with recent evidence (Debevec et al., 2017), we suggest that moderate physical activity can attenuate hypoxia-induced oxidative stress, as shown by the glutathione redox status.

Plasma FFA levels reflect the rate of adipose tissue lipolysis. Evidence indicates that hypoxia decreases lipid mobilization and FFA levels, while bed rest has opposite effects (Barbe et al., 1998; de Glisezinski et al., 1999). In addition, oxidative stress may directly activate lipolysis, while administration of antioxidants may reduce FFA release (Krawczyk et al., 2012). Our results are in agreement with the above findings, hypoxia decreased FFA levels, although this is confirmed only for subjects in ambulatory condition and not in bedridden volunteers (Jakobsson and Jorfeldt, 1990; Burtscher, 2013). Thus, lipolysis may be down-regulated by chronic hypoxia in physically active subjects. Such inhibitory effect may be blunted during physical inactivity due to increased oxidative stress.

### Study Limitations

The main limitation of the present study is the short duration of interventions. Exposition to 10 days of hypoxia may not allow a full development of inflammatory and metabolic alterations. It may be possible that a more prolonged exposition to hypoxia and/or bed rest could better mimic pathophysiology of chronic disease conditions. Nonetheless, the main study goal was to explore the interaction between hypoxia and bed rest on several biomarkers. In addition, we recognize the fact that only 11 male volunteers were enrolled. Finally, we recognize that the definitions of biomarkers as pro-atherogenic and anti-atherogenic are speculative and were not directly demonstrated in the present study through assessment of cardiovascular/autonomic changes.

## Conclusion

We found that experimental normobaric hypoxia in healthy volunteers was associated with an atherogenic pattern of metabolic and inflammatory markers. Nonetheless, selected anti-atherosclerotic pathways are activated in parallel, especially when decreased oxygen availability is combined with moderate level of physical activity, as opposed to enforced bed rest. These results provide mechanisms underlying the strict association between hypoxic diseases and cardiovascular complications (Roversi et al., 2014). In addition, our results may support the notion that healthy and physically active individuals living at high altitude may have protective effects on cardiovascular diseases (Faeh et al., 2009; Burtscher, 2013). The positive effects observed when moderate physical activity is associated with hypoxia is relevant for clinical application. Our results should be taken into account when physical exercise and rehabilitation programs are prescribed to patients with cardiopulmonary pathologies.

## Author Contributions

GB: research design, data analysis and statistical analysis, manuscript draft and had primary responsibility for final content. FDG: data analysis and interpretation, statistical analysis, manuscript draft, and research conduction. AM: research conduction. NF: data analysis and interpretation, statistical analysis, and manuscript draft. FM: manuscript draft. RS: data analysis and interpretation, and manuscript draft. AG: data analysis, manuscript draft. BD: sample analysis. MG: data analysis and interpretation. ML, GZ, and PS: data analysis and interpretation, manuscript draft. GG: data analysis and interpretation. IM: research design and manuscript draft. All authors have read and approved the final manuscript.

## Conflict of Interest Statement

The authors declare that the research was conducted in the absence of any commercial or financial relationships that could be construed as a potential conflict of interest.

## Abbreviations

ANCOVA: analysis of co-variance
ANOVA: analysis of variance
ASR: absolute synthesis rate
CETP: cholesterylester transfer protein
COPD: chronic obstructive pulmonary disease
CRP: C-reactive protein
DHA: docosahexaenoic acid
DXA: dual-energy X-ray absorptiometry
EPA: eicosapentaenoic acid
FFA: free-fatty acids
FSR: fractional synthesis rate
GCL-C: catalytic subunits of glutamate-cysteine ligase
GCL-M: modulatory subunits of glutamate-cysteine ligase
GC-MS: gas chromatography–mass spectrometry
GSH/GSSG: reduced-to-oxidized glutathione ratio
HDL-C: high-density lipoprotein cholesterol
HL: hepatic lipase
HOMA2-IR: Homeostatic Model Assessment 2 of Insulin Resistance
LCAT: lecithin–cholesterol acyltransferase
LDL-C: low-density lipoprotein cholesterol
LPL: lipoprotein lipase
OSAS: obstructive sleep apnea syndrome
PUFA: polyunsaturated fatty acids
SAA: serum amyloid A
TRAIL: TNF-related apoptosis-inducing ligand

## References

[B1] Agostini F. Dalla Libera L. Rittweger J. Mazzucco S. Jurdana M. Mekjavic I. B. (2010). Effects of inactivity on human muscle glutathione synthesis by a double-tracer and single-biopsy approach. *J. Physiol.* 588(Pt 24) 5089–5104. 10.1113/jphysiol.2010.198283 20962001 PMC3036199

[B2] Ashfaq S. Abramson J. L. Jones D. P. Rhodes S. D. Weintraub W. S. Hooper W. C. (2006). The relationship between plasma levels of oxidized and reduced thiols and early atherosclerosis in healthy adults. *J. Am. Coll. Cardiol.* 47 1005–1011. 10.1016/j.jacc.2005.09.063 16516085

[B3] Avila M. A. Carretero M. V. Rodriguez E. N. Mato J. M. (1998). Regulation by hypoxia of methionine adenosyltransferase activity and gene expression in rat hepatocytes. *Gastroenterology* 114 364–371. 10.1016/S0016-5085(98)70489-5 9453498

[B4] Barbe P. Galitzky J. De Glisezinski I. Riviere D. Thalamas C. Senard J. M. (1998). Simulated microgravity increases beta-adrenergic lipolysis in human adipose tissue. *J. Clin. Endocrinol. Metab.* 83 619–625. 9467583 10.1210/jcem.83.2.4557

[B5] Biolo G. Agostini F. Simunic B. Sturma M. Torelli L. Preiser J. C. (2008). Positive energy balance is associated with accelerated muscle atrophy and increased erythrocyte glutathione turnover during 5 wk of bed rest. *Am. J. Clin. Nutr.* 88 950–958. 10.1093/ajcn/88.4.950 18842781

[B6] Biolo G. Ciocchi B. Stulle M. Piccoli A. Lorenzon S. Dal Mas V. (2005a). Metabolic consequences of physical inactivity. *J. Ren. Nutr.* 15 49–53. 10.1053/j.jrn.2004.09.009 15648007

[B7] Biolo G. Massolino B. Di Girolamo F. G. Fiotti N. Mearelli F. Mazzucco S. (2018). Intensive insulin therapy increases glutathione synthesis rate in surgical ICU patients with stress hyperglycemia. *PLoS One* 13:e0190291. 10.1371/journal.pone.0190291 29300728 PMC5754081

[B8] Biolo G. Secchiero P. De Giorgi S. Tisato V. Zauli G. (2012). The energy balance positively regulates the levels of circulating TNF-related apoptosis inducing ligand in humans. *Clin. Nutr.* 31 1018–1021. 10.1016/j.clnu.2012.04.016 22633079

[B9] Biolo G. Zorat F. Antonione R. Ciocchi B. (2005b). Muscle glutamine depletion in the intensive care unit. *Int. J. Biochem. Cell Biol.* 37 2169–2179. 16084750 10.1016/j.biocel.2005.05.001

[B10] Blaise S. Alberto J. M. Nédélec E. Ayav A. Pourié G. Bronowicki J. P. (2005). Mild neonatal hypoxia exacerbates the effects of vitamin-deficient diet on homocysteine metabolism in rats. *Pediatr. Res.* 57 777–782. 10.1203/01.PDR.0000161406.19231.98 15845641

[B11] Bosutti A. Malaponte G. Zanetti M. Castellino P. Heer M. Guarnieri G. (2008). Calorie restriction modulates inactivity-induced changes in the inflammatory markers C-reactive protein and pentraxin-3. *J. Clin. Endocrinol. Metab.* 93 3226–3229. 10.1210/jc.2007-1684 18492758

[B12] Bruning U. Fitzpatrick S. F. Frank T. Birtwistle M. Taylor C. T. Cheong A. (2012). NFκB and HIF display synergistic behaviour during hypoxic inflammation. *Cell. Mol. Life Sci.* 69 1319–1329. 10.1007/s00018-011-0876-2 22068612 PMC11114791

[B13] Burtscher M. (2013). Effects of living at higher altitudes on mortality: a narrative review. *Aging Dis.* 5 274–280. 10.14336/AD.2014.0500274 25110611 PMC4113517

[B14] Caglar F. N. T. Isiksacan N. Biyik I. Opan S. Cebe H. Akturk I. F. (2017). Presepsin (sCD14-ST): could it be a novel marker for the diagnosis of ST elevation myocardial infarction? *Arch. Med. Sci. Atheroscler. Dis.* 2 e3–e8. 10.5114/amsad.2017.66827 28905041 PMC5596112

[B15] Camont L. Chapman M. J. Kontush A. (2011). Biological activities of HDL subpopulations and their relevance to cardiovascular disease. *Trends Mol. Med.* 17 594–603. 10.1016/j.molmed.2011.05.013 21839683

[B16] Chatterjee C. Sparks D. L. (2011). Hepatic lipase, high density lipoproteins, and hypertriglyceridemia. *Am. J. Pathol.* 178 1429–1433. 10.1016/j.ajpath.2010.12.050 21406176 PMC3078429

[B17] Chenevier-Gobeaux C. Borderie D. Weiss N. Mallet-Coste T. Claessens Y. E. (2015). Presepsin (sCD14-ST), an innate immune response marker in sepsis. *Clin. Chim. Acta* 450 97–103. 10.1016/j.cca.2015.06.026 26164388

[B18] Daneshmand R. Kurl S. Tuomainen T. P. Virtanen J. K. (2017). Associations of estimated Δ-5-desaturase and Δ-6-desaturase activities with stroke risk factors and risk of stroke: the Kuopio Ischaemic heart disease risk factor study. *Br. J. Nutr.* 117 582–590. 10.1017/S000711451700054X 28382895

[B19] de Glisezinski I. Crampes F. Harant I. Havlik P. Gardette B. Jammes Y. (1999). Decrease of subcutaneous adipose tissue lipolysis after exposure to hypoxia during a simulated ascent of Mt Everest. *Pflugers Arch.* 439 134–140. 10.1007/s004240051137 10651010

[B20] Debevec T. McDonnell A. C. Macdonald I. A. Eiken O. Mekjavic I. B. (2014). Whole body and regional body composition changes following 10-day hypoxic confinement and unloading-inactivity. *Appl. Physiol. Nutr. Metab.* 39 386–395. 10.1139/apnm-2013-0278 24552383

[B21] Debevec T. Millet G. P. Pialoux V. (2017). Hypoxia-induced oxidative stress modulation with physical activity. *Front. Physiol.* 8:84. 10.3389/fphys.2017.00084 28243207 PMC5303750

[B22] Eltzschig H. K. Carmeliet P. (2011). Hypoxia and inflammation. *N. Engl. J. Med.* 364 656–665. 10.1056/NEJMra0910283 21323543 PMC3930928

[B23] Faeh D. Gutzwiller F. Bopp M. Swiss National Cohort Study Group (2009). Lower mortality from coronary heart disease and stroke at higher altitudes in Switzerland. *Circulation* 120 495–501. 10.1161/CIRCULATIONAHA.108.819250 19635973

[B24] Forde H. Harper E. Davenport C. Rochfort K. D. Wallace R. Murphy R. P. (2016). The beneficial pleiotropic effects of tumour necrosis factor-related apoptosis-inducing ligand (TRAIL) within the vasculature: a review of the evidence. *Atherosclerosis* 247 87–96. 10.1016/j.atherosclerosis.2016.02.002 26878368

[B25] Gortan Cappellari G. Losurdo P. Mazzucco S. Panizon E. Jevnicar M. Macaluso L. (2013). Treatment with n-3 polyunsaturated fatty acids reverses endothelial dysfunction and oxidative stress in experimental menopause. *J. Nutr. Biochem.* 24 371–379. 10.1016/j.jnutbio.2012.07.012 23159066

[B26] Harris W. S. Del Gobbo L. Tintle N. L. (2017). The omega-3 Index and relative risk for coronary heart disease mortality: estimation from 10 cohort studies. *Atherosclerosis* 262 51–54. 10.1016/j.atherosclerosis.2017.05.007 28511049

[B27] Hasokawa M. Shinohara M. Tsugawa H. Bamba T. Fukusaki E. Nishiumi S. (2012). Identification of biomarkers of stent restenosis with serum metabolomic profiling using gas chromatography/mass spectrometry. *Circ. J.* 76 1864–1873. 10.1253/circj.CJ-11-0622 22664753

[B28] Huang Z. Song L. Wang C. Liu J. Q. Chen C. (2011). Hypoxia-ischemia upregulates TRAIL and TRAIL receptors in the immature rat brain. *Dev. Neurosci.* 33 519–530. 10.1159/000334475 22286051

[B29] Jakobsson E. J. Jorfeldt L. (1990). Blood fuel metabolites at rest and during exercise in patients with advanced chronic obstructive pulmonary disease with and without chronic respiratory failure. *Respiration* 57 304–309. 10.1159/000195861 2126638

[B30] Jezková J. Nováková O. Kolár F. Tvrzická E. Neckár J. Novák F. (2002). Chronic hypoxia alters fatty acid composition of phospholipids in right and left ventricular myocardium. *Mol. Cell. Biochem.* 232 49–56. 10.1023/A:1014889115509 12030379

[B31] Kim J. Y. Lee E. Y. Park J. K. Song Y. W. Kim J. R. Cho K. H. (2016). Patients with rheumatoid arthritis show altered lipoprotein profiles with dysfunctional high-density lipoproteins that can exacerbate inflammatory and atherogenic process. *PLoS One* 11:e0164564. 10.1371/journal.pone.0164564 27736980 PMC5063466

[B32] Krawczyk S. A. Haller J. F. Ferrante T. Zoeller R. A. Corkey B. E. (2012). Reactive oxygen species facilitate translocation of hormone sensitive lipase to the lipid droplet during lipolysis in human differentiated adipocytes. *PLoS One* 7:e34904. 10.1371/journal.pone.0034904 22493722 PMC3321042

[B33] Levy J. C. Matthews D. R. Hermans M. P. (1998). Correct homeostasis model assessment (HOMA) evaluation uses the computer program. *Diabetes Care* 21 2191–2192. 10.2337/diacare.21.12.2191 9839117

[B34] Liu H. Yang E. Lu X. Zuo C. He Y. Jia D. (2015). Serum levels of tumor necrosis factor-related apoptosis-inducing ligand correlate with the severity of pulmonary hypertension. *Pulm. Pharmacol. Ther.* 33 39–46. 10.1016/j.pupt.2015.06.002 26086178

[B35] Lu Y. Vaarhorst A. Merry A. H. Dollé M. E. Hovenier R. Imholz S. (2012). Markers of endogenous desaturase activity and risk of coronary heart disease in the CAREMA cohort study. *PLoS One* 7:e41681. 10.1371/journal.pone.0041681 22911844 PMC3402436

[B36] Mazzucco S. Agostini F. Biolo G. (2010a). Inactivity-mediated insulin resistance is associated with upregulated pro-inflammatory fatty acids in human cell membranes. *Clin. Nutr.* 29 386–390. 10.1016/j.clnu.2009.09.006 19875212

[B37] Mazzucco S. Agostini F. Mangogna A. Cattin L. Biolo G. (2010b). Prolonged inactivity up-regulates cholesteryl ester transfer protein independently of body fat changes in humans. *J. Clin. Endocrinol. Metab.* 95 2508–2512. 10.1210/jc.2009-2561 20228163

[B38] McEneny J. McKavanagh P. York E. Nadeem N. Harbinson M. Stevenson M. (2016). Serum- and HDL3-serum amyloid A and HDL3-LCAT activity are influenced by increased CVD-burden. *Atherosclerosis* 244 172–178. 10.1016/j.atherosclerosis.2015.11.018 26647373

[B39] Moriyama K. Takahashi E. (2016). HDL2/HDL3 ratio changes, metabolic syndrome markers, and other factors in a Japanese population. *J. Atheroscler. Thromb.* 23 704–712. 10.5551/jat.32896 26686740 PMC7399289

[B40] Nakano D. Hayashi T. Tazawa N. Yamashita C. Inamoto S. Okuda N. (2005). Chronic hypoxia accelerates the progression of atherosclerosis in apolipoprotein E-knockout mice. *Hypertens. Res.* 28 837–845. 10.1291/hypres.28.837 16471178

[B41] Nygård O. Nordrehaug J. E. Refsum H. Ueland P. M. Farstad M. Vollset S. E. (1997). Plasma homocysteine levels and mortality in patients with coronary artery disease. *N. Engl. J. Med.* 337 230–236. 10.1056/NEJM199707243370403 9227928

[B42] Pan D. A. Lillioja S. Milner M. R. Kriketos A. D. Baur L. A. Bogardus C. (1995). Skeletal muscle membrane lipid composition is related to adiposity and insulin action. *J. Clin. Invest.* 96 2802–2808. 10.1172/JCI118350 8675650 PMC185990

[B43] Pišot R. Marusic U. Biolo G. Mazzucco S. Lazzer S. Grassi B. (1985). Greater loss in muscle mass and function but smaller metabolic alterations in older compared with younger men following 2 wk of bed rest and recovery. *J. Appl. Physiol.* 120 922–929. 10.1152/japplphysiol.00858.2015 26823343

[B44] Qi L. Qi Q. Prudente S. Mendonca C. Andreozzi F. di Pietro N. (2013). Association between a genetic variant related to glutamic acid metabolism and coronary heart disease in individuals with type 2 diabetes. *JAMA* 310 821–828. 10.1001/jama.2013.276305 23982368 PMC3858847

[B45] Rong D. Liu J. Jia X. Al-Nafisee D. Jia S. Sun G. (2017). Hyperhomocysteinaemia is an independent risk factor for peripheral arterial disease in a Chinese Han population. *Atherosclerosis* 263 205–210. 10.1016/j.atherosclerosis.2017.05.006 28651188 PMC5557685

[B46] Roversi S. Roversi P. Spadafora G. Rossi R. Fabbri L. M. (2014). Coronary artery disease concomitant with chronic obstructive pulmonary disease. *Eur. J. Clin. Invest.* 44 93–102. 10.1111/eci.12181 24164255

[B47] Salazar A. Maña J. Pinto X. Argimon J. M. Castiñeiras M. J. Fiol C. (1998). Low levels of high density lipoprotein cholesterol in patients with active sarcoidosis. *Atherosclerosis* 136 133–137. 10.1016/S0021-9150(97)00198-6 9580477

[B48] Sasaki N. Yamashita T. Takeda M. Hirata K. (2012). Regulatory T cells in atherogenesis. *J. Atheroscler. Thromb.* 19 503–515. 10.5551/jat.10934 22498766

[B49] Simpson E. J. Debevec T. Eiken O. Mekjavic I. Macdonald I. A. (1985). PlanHab: the combined and separate effects of 16 days of bed rest and normobaric hypoxic confinement on circulating lipids and indices of insulin sensitivity in healthy men. *J. Appl. Physiol.* 120 947–955. 10.1152/japplphysiol.00897.2015 26769956 PMC4835909

[B50] Volpato S. Ferrucci L. Secchiero P. Corallini F. Zuliani G. Fellin R. (2011). Association of tumor necrosis factor-related apoptosis-inducing ligand with total and cardiovascular mortality in older adults. *Atherosclerosis* 215 452–458. 10.1016/j.atherosclerosis.2010.11.004 21122855 PMC3070040

[B51] Voltan R. Secchiero P. Casciano F. Milani D. Zauli G. Tisato V. (2016). Redox signaling and oxidative stress: cross talk with TNF-related apoptosis inducing ligand activity. *Int. J. Biochem. Cell Biol.* 81(Pt B) 364–374. 10.1016/j.biocel.2016.09.019 27686849

[B52] Watkins S. M. Hotamisligil G. S. (2012). Promoting atherosclerosis in type 1 diabetes through the selective activation of arachidonic acid and PGE(2) production. *Circ. Res.* 111 394–396. 10.1161/CIRCRESAHA.112.273508 22859668

[B53] Yanagibori R. Kondo K. Suzuki Y. Kawakubo K. Iwamoto T. Itakura H. (1998). Effect of 20 days’ bed rest on the reverse cholesterol transport system in healthy young subjects. *J. Intern. Med.* 243 307–312. 10.1046/j.1365-2796.1998.00303.x 9627145

[B54] Yang C. Chen J. Wu F. Li J. Liang P. Zhang H. (2014). Effects of 60-day head-down bed rest on osteocalcin, glycolipid metabolism and their association with or without resistance training. *Clin. Endocrinol.* 81 671–678. 10.1111/cen.12535 24975467

